# An extremely rare case with right superior pulmonary vein translocation

**DOI:** 10.1186/s40792-020-00860-7

**Published:** 2020-05-07

**Authors:** Go Kamimura, Kazuhiro Ueda, Koki Maeda, Masaya Aoki, Toshiyuki Nagata, Naoya Yokomakura, Masami Sato

**Affiliations:** Department of General Thoracic Surgery, Kagoshima University Graduate School of Dental and Medical Science, 8-35-1 Sakuragaoka, Kagoshima, 890-8520 Japan

**Keywords:** Thoracoscopic lobectomy, Lung cancer, Pulmonary vein, Three-dimensional images

## Abstract

**Background:**

There have been a number of reports on pulmonary venous anomalies. However, most of the reports focused on the anatomical branching pattern of the peripheral pulmonary veins.

**Case presentation:**

We report a 75-year-old female whose right superior pulmonary vein V1 existed dorsal to the right main pulmonary artery and V2+3 existed dorsal to V4+5. Thus, we could not find V1 and V2+3 in the hilum just after a thoracotomy to perform right upper lobectomy for lung cancer. Thus, the right main pulmonary artery and the superior trunk (A1+3) were exposed without cutting the superior pulmonary vein.

**Conclusion:**

There has been no report so far regarding this type of pulmonary vein translocation. Preoperative three-dimensional computed tomography images were helpful to identify this variant.

## Background

There have been some reports regarding the anatomical variations of pulmonary veins, particularly regarding branching patterns of peripheral pulmonary veins [1, 2]. Overlooking of the correct vessels can cause intraoperative accidental bleeding, while misunderstanding of the vessels, that should be dissected, can cause intractable complications such as pulmonary congestion. Therefore, preoperative accurate identification of bronchovasculatures on preoperative computed tomography (CT) is indispensable to avoid iatrogenic complications. We report an extremely rare case of translocation of the superior pulmonary vein, which was detected by preoperative three-dimensional (3D) volume rendering CT images, yielding safe dissection of the pulmonary vessels during right upper lobectomy.

## Case presentation

**Fig. 1:**
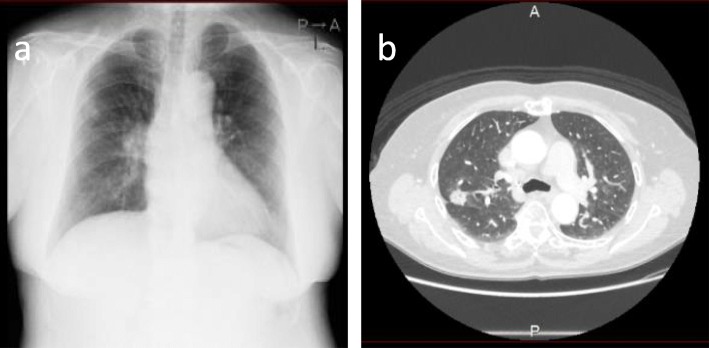
An abnormal chest shadow can be seen on a chest roentgenogram in the right upper lung field (**a**). A chest computed tomography scan showed an abnormal lesion, 2.6 × 2.4 cm in size, in the right upper lobe (**b**)

**Fig. 2:**
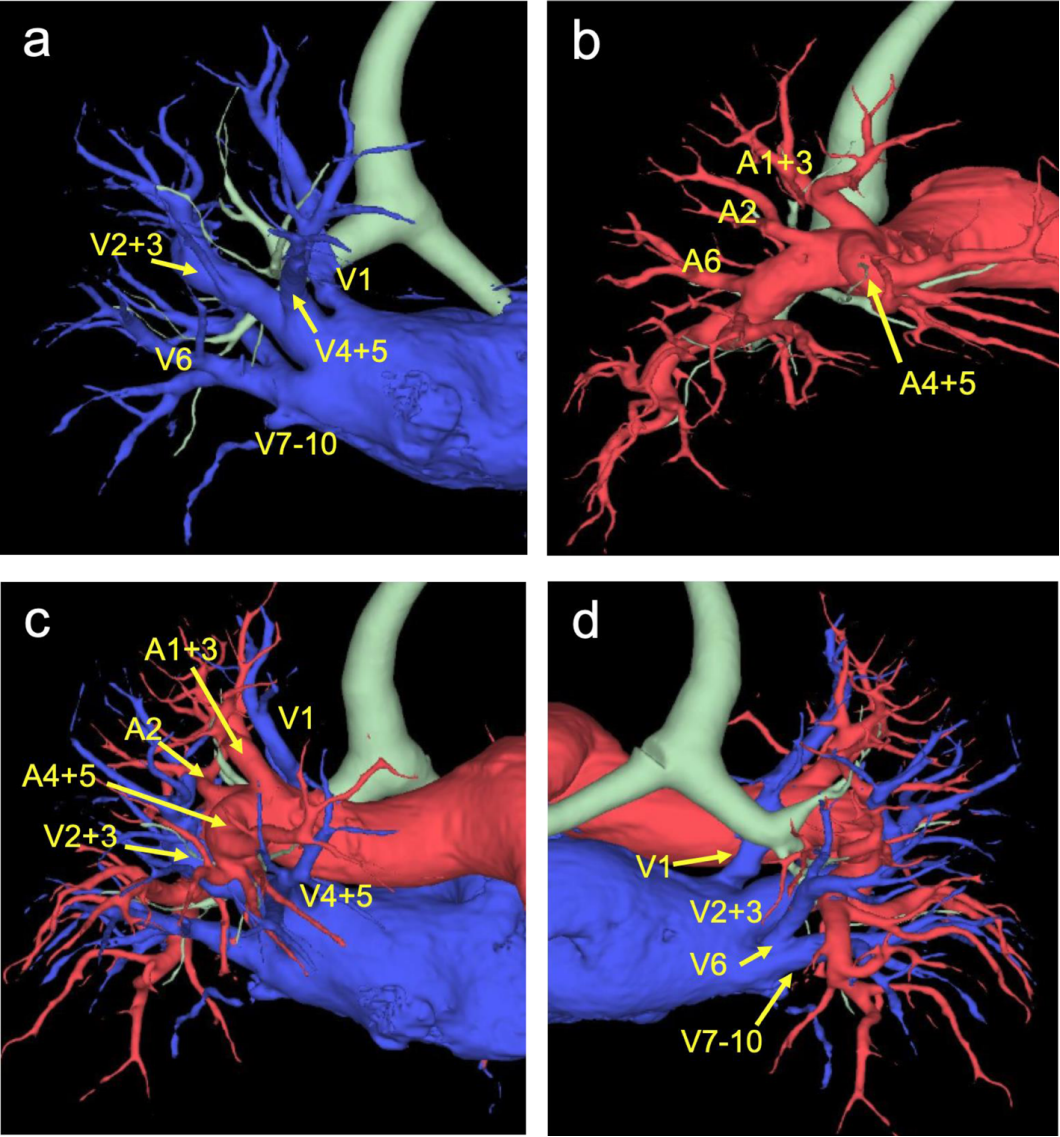
The whole image of the right pulmonary vein is observed with the 3D image. The segmental vein (V1) flowed into the left atrium alone. The segmental vein (V2+3) joins the segmental vein (V4+5) from the back of the segmental vein (V4+5) flows into the left atrium (**a**). In observation of the 3D image, the whole image of the pulmonary artery was as usual (**b**). At the hilum, the main trunk of the pulmonary artery is above the segmental veins (V1 and V2+3), but between the lobes, the segmental vein (V2+3) is over the main pulmonary artery (**c**). From the dorsal view, the segmental vein (V1) exists in front of the right upper lobe bronchus (**d**)

A 75-year-old female was referred to our hospital due to an abnormal shadow in the right upper lung field on a chest roentgenogram (Fig. 1a). The patient had no symptoms and any significant medical history except for asthma. A CT scan revealed an abnormal lesion measuring 3.8 × 3.0 cm (solid 3.5 × 2.7 cm) in size on pulmonary window settings (Fig. 1b). An 18F-fluorodeoxyglucose positron emission tomography (FDG-PET) showed abnormal uptake in the tumor with a maximum standardized uptake value (SUV max) of 3.0. These findings were compatible with lung carcinoma, clinical T2aN0M0, stage IB (ver.8 TNM system). Three-dimensional (3D)-volume rendering CT images, that was reconstructed by chest CT using commercially available software (Synapse Vincent, Fuji, Japan), showed that the right pulmonary vein V1 existed just behind the main part of the right pulmonary artery and V2+3 existed dorsal to V4+5 (Fig. 2a–c). Collectively, the pulmonary vein V1 was located between the pulmonary artery and the main bronchus in pulmonary hilum (Fig. 2d).

**Fig. 3:**
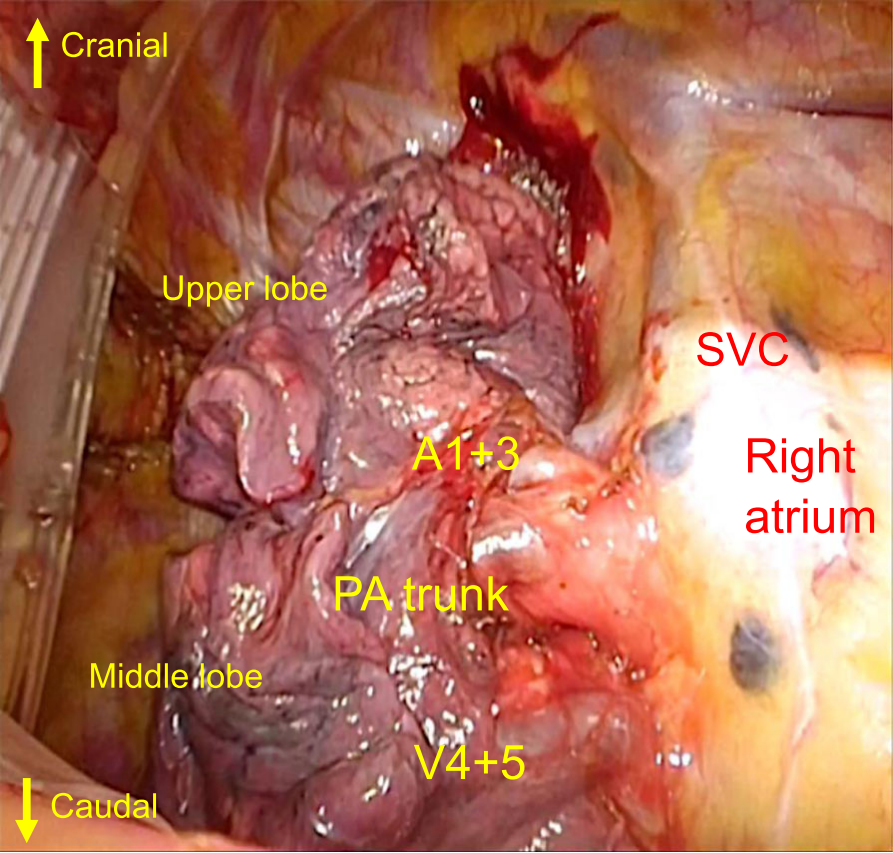
From the operation forward view, the segmental vein V1 and vein V2+3 cannot be seen in the usual pulmonary hilum position. Instead, the main pulmonary artery runs in front of the V1

Right upper lobectomy with combined resection of hilar and upper mediastinal lymph nodes was undertaken under the guidance with 3D bronchovasculature images. After an anterolateral thoracotomy, the pulmonary vein V1 and V2+3 was not detected in the pulmonary hilum via the anterolateral thoracotomy. Only the right main pulmonary artery and pulmonary vein V4+5 were detected (Fig. 3). Although we generally use an endstapler to divide the pulmonary vein V1–3, we could not use this procedure in this case because it was difficult to secure the necessary space to insert an endstapler behind the right main pulmonary artery. Therefore, dissection of the incomplete minor fissure was performed initially to facilitate anatomical identification of the upper lobe vasculatures: after ligating and cutting of the pulmonary vein V2+3, the main part of the pulmonary artery could be encircled, which helped visualization of V1. The pathological diagnosis was adenocarcinoma, pT2aN0M0, p-stage IB. She was discharged on postoperative day 9 and the postoperative course was uneventful to date.

Misdiagnosis of the pulmonary vein during major lung resection leads to severe complications, such as pulmonary congestion, pulmonary vein thrombus, or cerebral infarctions [3, 4]. Therefore, we should be careful when ligating the pulmonary veins during major lung resection. It is necessary to figure out the anatomical variations of pulmonary vein preoperatively. There are some surgical reports about lung cancer with the variant pulmonary veins. Yamashita reported anatomical variations of pulmonary veins of the upper lobe [5]. However, there have been no reports of pulmonary vein V1 existing dorsal to the right main pulmonary artery and V2+3 existing dorsal to V4+5.

Generally, three variations are well known: (A) the common duct of the superior and inferior pulmonary vein, (B) a segment vein flowing into the left atrium independently except for superior pulmonary vein and inferior pulmonary vein, and (C) the superior pulmonary vein and inferior pulmonary vein flowing into another position of the left atrium [6]. In those three types of variation, variant veins always run in front of the main pulmonary artery, in contrast to our case, except that aberrant V2 arises from the inferior pulmonary vein and lies dorsal to the main pulmonary artery.

According to literatures, there are two cases associated with right superior pulmonary vein translocation [7, 8]. In one patient, V2+V3a lay dorsal to the main pulmonary artery, while the remaining branch (V1+V3b) ran normally [7]. In another patient, the superior pulmonary vein (V1+2+3) ran between the main pulmonary artery and the upper lobe bronchus [8]. However, unlike our patient, both the superior pulmonary vein branches in these two patients appeared to exist cranial to A4+5. In addition, both patients had tracheal bronchus that is often accompanied by pulmonary vessel variations [9], although our patient did not have any anatomical variations of pulmonary bronchovasculatures or pulmonary lobulations other than pulmonary vein translocation.

We can detect anatomical variations of pulmonary veins and arteries in advance by the following four methods: (1) pulmonary artery angiography to visualize pulmonary arteries, (2) studying contrast-enhanced CT in detail, (3) detecting all pulmonary arteries and veins during a surgical operation, and (4) studying preoperative 3D images [10]. Although there is a limit to detect anatomical variations from the flat two-dimensional view, the 3D view helps us to visually detect anatomical variations more easily and rapidly as shown in our case. To avoid severe complications, we should pay attention to anatomical variations of pulmonary veins preoperatively. From these viewpoints, 3D images technologies help us to detect anatomical variations easily and can promote safer lung resection.

## Conclusions

We experienced an extremely rare case with the right superior pulmonary vein translocation. There has been no report so far regarding this type of pulmonary vein translocation. And 3D images technologies help us to detect anatomical variations easily and can promote safer lung resection.

## Data Availability

All related data are included within the article.

## Abbreviations

3D-CT: Three-dimensional volume rendering computed tomography
FDG-PET: 18F-fluorodeoxyglucose positron emission tomography
SUV max: Maximum standardized uptake value
